# Pickering Emulsion-Based Marbles for Cellular Capsules

**DOI:** 10.3390/ma9070572

**Published:** 2016-07-14

**Authors:** Guangzhao Zhang, Chaoyang Wang

**Affiliations:** Research Institute of Materials Science, South China University of Technology, Guangzhou 510640, China; msgzhzhang@mail.scut.edu.cn

**Keywords:** Pickering emulsion, liquid marbles, cellular capsule, drug release, biodegradable

## Abstract

The biodegradable cellular capsule, being prepared from simple vaporization of liquid marbles, is an ideal vehicle for the potential application of drug encapsulation and release. This paper reports the fabrication of cellular capsules via facile vaporization of Pickering emulsion marbles in an ambient atmosphere. Stable Pickering emulsion (water in oil) was prepared while utilizing dichloromethane (containing poly(l-lactic acid)) and partially hydrophobic silica particles as oil phase and stabilizing agents respectively. Then, the Pickering emulsion marbles were formed by dropping emulsion into a petri dish containing silica particles with a syringe followed by rolling. The cellular capsules were finally obtained after the complete vaporization of both oil and water phases. The technique of scanning electron microscope (SEM) was employed to research the microstructure and surface morphology of the prepared capsules and the results showed the cellular structure as expected. An in vitro drug release test was implemented which showed a sustained release property of the prepared cellular capsules. In addition, the use of biodegradable poly(l-lactic acid) and the biocompatible silica particles also made the fabricated cellular capsules of great potential in the application of sustained drug release.

## 1. Introduction

It is well known that the term ‘liquid marble’ is defined to describe aqueous droplets which are enwrapped by self-organized hydrophobic powders [1,2]. Since Aussillous transported liquid marble via rolling water droplets across hydrophobic grains [3], a large number of researchers have focused on the investigation of liquid marbles [4,5,6]. In fact, the liquid marbles had already existed for a long time, if one considers rainwater dropping into dry dirt or the water droplets falling into large quantity of wheat flour, although few people had previously paid attention to it. Due to their distinctive properties—including the ability to be divided or fused together with self-recovery enwrapped layers [7], low frictional resistance derived from small a contact area with the subsurface [8] as well as relatively facile manipulation—extensive research on liquid marbles has been carried out recently. Moreover, these unique properties make investigation of liquid marbles not limited only to theoretical research, but also extended to some practical applications, such as miniature or micro-chemical reactors [9], sensors [10], pollution detection [11], vehicles for transporting microfluidics [12], and oil spill treatment [13]. For instance, Arbatan and coworkers employed the hydrophobic powder precipitated calcium carbonate (PCC) to prepare the ‘blood liquid marbles’ as micro-reactors for biological reactions and diagnostic experiments [14]. Interestingly, the blood marbles they reported could identify the blood type rapidly while showing some advantages—including low cost, which free medical facilities rely on, and disposability—when compared with conventional blood typing techniques. Another interesting example worthy of mention is Fujii et al.’s work [15]: They reported a smart liquid marble which could move on the surface of water under the control of near-infrared laser or sunlight. Moreover, the marbles they prepared could also release the encapsulated material at a specific time and place by controllable external stimuli, making it an ideal candidate for target controlled release. In addition, other explorations of liquid marbles for chemical synthesis, mass transport and self-assembly have been reported recently [16], suggesting promising potential applications of liquid marbles.

Similar to, but different from liquid marbles, Pickering emulsion also makes use of solid particles to absorb to a liquid–liquid interface, which leads to the formation of emulsion and sequentially keeps it stable. The biggest difference lies in the medium surrounding the emulsion droplets being a liquid phase (water or oil), while the encapsulating medium is replaced by air for liquid marbles [17]. The amphiphilic solid particles used for the stabilization of Pickering emulsion exhibit low toxicity and favorable biocompatibility when compared with traditional emulsion stabilized with harmful surfactant, which makes the Pickering emulsion an environmentally friendly candidate for applications in the fields of food [18], cosmetics [19], and green catalysis [20]. In view of the flexibility in the selection of solid particles and oil phase, there is nothing surprising about the fact that Pickering emulsion has drawn much attention during the last decades. Additionally, the unique property of Pickering emulsion templates was also explored fully by researchers to prepare porous material [21] or cellular structure [22], further expanding its practical applications. 

**Scheme 1:**
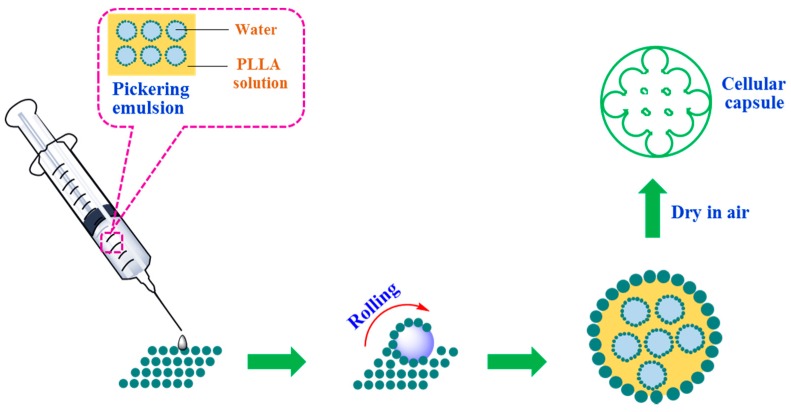
The schematic illustration of the preparation of Pickering emulsion-based marbles and cellular capsules.

Inspired by the pioneer’s work, we combined the merits of liquid marbles and Pickering emulsion in this report, and prepared the Pickering emulsion-based marbles to fabricate cellular capsules via a facile rolling method. To date, although a considerable amount of research on liquid marbles has been undertaken, examples of exploiting Pickering emulsion as an encapsulated liquid to prepare emulsion marbles are rare. The design of our work is presented in detail in Scheme 1: The water in oil Pickering emulsion was prepared first using dichloromethane (containing poly(l-lactic acid)) as the oil phase; then the fabricated emulsion was transferred drop by drop into a petri dish containing sufficient solid particles, using a syringe equipped with a 26 gauge needle; followed by immediate rolling to form stable emulsion-based marbles. The fabricated emulsion marbles were transported to a glass substrate and then dried in air at room temperature. The cellular capsules were obtained after the complete evaporation of both dichloromethane and water. Influences on the formation of emulsion marbles, including the concentration of polymer, stabilizer particle content and internal phase volume fraction were studied amply, and the technique of scanning electron microscopy (SEM) was also utilized to characterize the microstructure and morphology of the prepared capsules. Moreover, an in vitro drug release test was also carried out and the results suggest that the fabricated capsule is a candidate for sustained drug release.

## 2. Materials and Methods

### 2.1. Materials

Poly(l-lactic acid) (PLLA, molecular weight *M*_w_ = 200,000 g/mol) was purchased from Shandong Medical Instrument Research Institute (Jinan, China). Partially hydrophobic silica (H_30_) (with an average diameter of 20 nm and 50% of surface hydroxyl content) was obtained from Wacker Chemie Company (Munich, Germany). Dichloromethane (CH_2_Cl_2_) was purchased from Guangzhou Chemical Factory (Guangzhou, China). Enrofloxacin (98%) was bought from J&K Scientific Co., Ltd. (Shanghai, China). Water used in all experiments was purified with a Millipore purification apparatus (Boston, MA, USA) with a resistance higher than 18.0 MΩ·cm.

### 2.2. Preparation of Pickering Emulsion

The water in oil Pickering emulsion was prepared according to the recipes listed in Table 1. Typically, a certain amount of PLLA was dissolved in dichloromethane (CH_2_Cl_2_) to form a homogeneous PLLA solution. Then, the partially hydrophobic silica particles (H_30_) were uniformly dispersed into PLLA solution with the assistance of sonication. After that, ultrapure water was added to the dispersion followed by emulsifying with a vortex mixer (Fisher Scientific, Haimen, China) at 3000 rpm for 3 min. It was noted that the sample P_0-0.1_ was just a comparison which was prepared by dissolving 0.10 g of PLLA into 2 mL of CH_2_Cl_2_.

### 2.3. Preparation of Pickering Emulsion-Based Marbles and Cellular Capsules

The general preparation method of Pickering emulsion-based marbles is described as follows. An adequate amount of as-prepared Pickering emulsion was loaded to a 1 mL syringe equipped with a 26-gauge needle at first. Then, the loaded emulsion was injected dropwise into a petri dish containing H_30_ silica powder followed by vigorously shaking the dish in a horizontal direction, keeping the emulsion droplet rolling until its surface was totally covered by silica particles. The Pickering emulsion marbles were obtained after removing residual silica powder. For the fabrication of cellular capsules, the only procedure which needed to be implemented was to place the prepared Pickering emulsion marbles in air at an ambient temperature for 48 h for the complete vaporization of both oil (CH_2_Cl_2_) and water phases.

### 2.4. In Vitro Drug Release

To fabricate the drug-loaded cellular structure, 1 mg of enrofloxacin was dissolved into 2 mL of CH_2_Cl_2_ solution alongside the PLLA prior to adding H_30_ particles, and the following procedure was identical to the description in Section 2.3 above. The in vitro drug release investigation was implemented by measuring the absorbance of released enrofloxacin at 271 nm with an Ultraviolet–visible (UV−vis) spectrophotometer (U-3010, Hitachi, Tokyo, Japan). For this experiment, typically 20 mg (containing 200 μg of enrofloxacin) of cellular capsule was placed into a glass bottle containing 20 mL of ultrapure water. Then, the bottle was incubated in a shaking incubator at 37 °C for release. 2 mL of release medium was withdrawn and equal fresh ultrapure water was added as a substitute at predetermined time intervals. The collected aqueous medium was analyzed by a UV–vis technique according to the calibration curves of enrofloxacin established from standard enrofloxacin solution. The assay was performed in triplicate.

### 2.5. Characterization

The Pickering emulsion droplets were observed with an optical microscope (Carl Zeiss, Oberkochen, Germany) equipped with a digital camera. The morphology of cellular capsule was characterized with a Zeiss EVO 18 scanning electron microscope (SEM) (Oberkochen, Germany). Generally, capsule samples were stuck onto conductive tape prior to gold coating with a sputter coater, followed by observation under a 10 kV acceleration voltage.

### 2.6. Estimation of H_30_ Particles Content Using for Stabilizing Emulsion Marbles

In order to estimate the content of H_30_ particles which serve to stabilize emulsion marbles, we set the volume of each Pickering emulsion droplet as 20 μL and measured the mass of ten dried capsules which was 11.6 mg. Then, 200 μL of Pickering emulsion was placed in a glass bottle in an ambient atmosphere for the complete vaporization of CH_2_Cl_2_ and water. After that, the mass of the residual scaffold was also measured and the result was 10.8 mg. Therefore, the average content (*W_a_*) of H_30_ particles used for stabilizing emulsion marbles was:

*W_a_* = (11.6 − 10.8)/10 = 0.08 mg

## 3. Result and Discussion

### 3.1. Preparation of Pickering Emulsion

**Figure 1:**
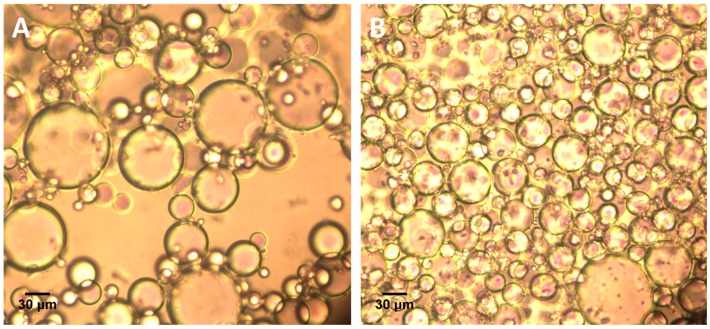
Optical microscope image of prepared Pickering emulsion with different H_30_ concentrations: (**A**) 1 w/v % of H_30_ silica particles, sample P_0.02-0.1-2_; (**B**) 2 w/v % of H_30_ silica particles, sample P_0.04-0.1-2_.

The Pickering emulsion was prepared via simple emulsifying of the mixture of PLLA solution and water with the help of H_30_ stabilizer. It was observed that the concentration of H_30_ silica particles have a significant influence on the stability of prepared Pickering emulsion. We set the volume of oil and water phase at 2 mL respectively and varied the amount of H_30_ silica particles for detailed research in our experiments (Table 1). It was found that when the concentration of H_30_ particles was 0.5 w/v % (sample P_0.01-0.1-2_, with respect of oil phase, and similarly from here), stratification appeared in the formed Pickering emulsion after it was placed in an ambient atmosphere for 48 h, which suggested that 0.5 w/v % of H_30_ silica particles was not enough for the formation of a stable Pickering emulsion. In fact, it was too difficult to observe the morphology of these Pickering emulsion droplets with optical microscope in conjunction with the volatility of CH_2_Cl_2_. However, when the concentration of H_30_ particles were increased to 1 w/v % and 2 w/v % (P_0.02-0.1-2_ and P_0.04-0.1-2_), the formed emulsion remained stable for 2 days. An optical microscope was employed for the observation of formed Pickering emulsion droplets, and the result is shown in Figure 1. As an intuitive demonstration, the droplets’ size distribution was also analyzed and the results are exhibited in Figure S1. It was clear that the size of the emulsion droplets decreased dramatically with the increase of H_30_ stabilizer, which accorded with the normal emulsion stabilization mechanism. The concentration of PLLA in CH_2_Cl_2_ did not show significant influence on the stability of emulsion in our experiments, but did increase the viscosity of formed emulsion. When raising the concentration of PLLA from 5 w/v % to 6 w/v % (P_0.04-0.5-2_), the viscosity of emulsion increased apparently and became difficult to inject dropwise by syringe (Figure S2A). It should be noted that the increase of internal volume fraction (P_0.04-0.5-3_) also made the formed emulsion more viscous, which resulted in the same phenomenon as the increment of PLLA. To determine the type of Pickering emulsion, we carried out a simple test by dropping the formed emulsion into a CH_2_Cl_2_ solution and water respectively. As shown in Figure S3, the emulsion droplets were destroyed and spread out in CH_2_Cl_2_, while remaining stable in water, indicating that the formed emulsion was water in oil type (W/O).

### 3.2. Preparation of Pickering Emulsion Marbles and Cellular Capsule

**Figure 2:**
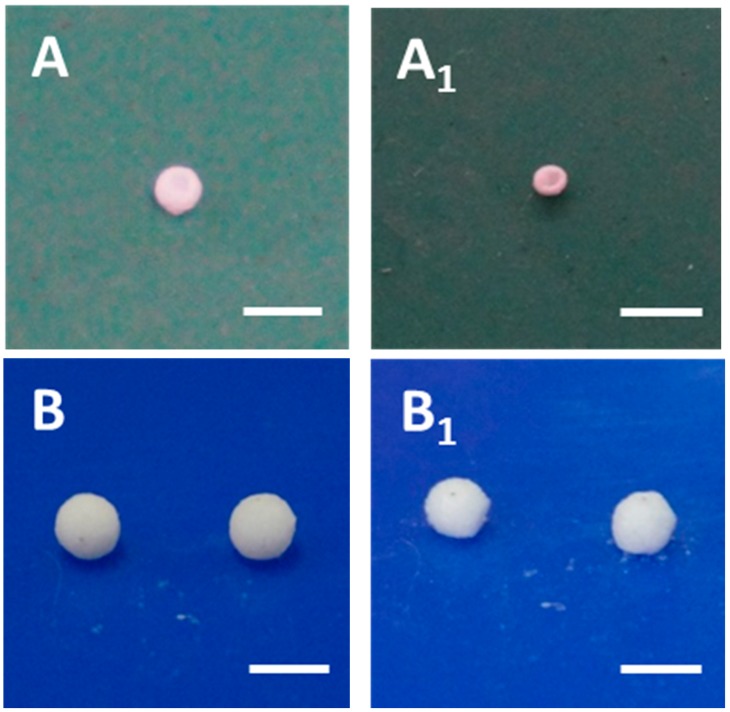
Digital photographs of prepared liquid marbles: (**A**) liquid marble directly deriving from PLLA solution; (**A_1_**) the formed capsule after the vaporization of dichloromethane; (**B**) Pickering emulsion marbles; (**B_1_**) the formed cellular capsule after the vaporization of both oil and water phase. All scale bars show 3 mm.

It is well known that the adhesivity of powder to a specific liquid is vital for the formation of stable liquid marbles. Considering the fact that the Pickering emulsion was water in oil type, partially hydrophobic silica powder (H_30_) was chosen to cover the surface of Pickering emulsion droplets for the preparation of emulsion marbles. It was found that the stability of Pickering emulsion was of great significance to the formation of emulsion marbles. When the concentration of stabilizer particle (H_30_) was 0.5 w/v %, the formed emulsion marbles remained intact in a petri dish containing a large amount of silica powder, but broke up immediately after transfer to a glass base (Figure S2C). This phenomenon suggested that the Pickering emulsion in an unstable state was difficult to cover with powder for forming stable emulsion marbles. In fact, the unstable Pickering emulsion should separate to form the initial oil and water phase from a uniform state according to the Ostwald Ripening theory, resulting in direct contact of external H_30_ powder with split water. Due to the hydrophobic property of H_30_ silica powder, it was difficult to adhere it to the surface of a water droplet for the formation of stable liquid marbles. In order to confirm our speculation, an experiment using pure water to prepare liquid marbles with H_30_ was carried out and the results are shown in Figure S4. Obviously, although the marble remained stable in large qualities of H_30_ powders, it collapsed immediately after transfer onto a glass subtrate. When the concentration of H_30_ particles was increased to 1 w/v %, the formed emulsion marbles became more stable than at 0.5 w/v %. However, an unexpected phenomenon appeared—the water droplet was squeezed out from one side of the marble (Figure S2D,E) during the drying process. Considering the fact that the evaporation rate of CH_2_Cl_2_ was much faster than that of water at room temperature, partial demulsification appeared in the formed marble during the vaporization of oil phase, resulting in the extrusion of the split water droplet from one side of the marble. When increasing the concentration of particles to 2 w/v %, the Pickering emulsion formed spherical and stable marbles (Figure 2B), and the formed emulsion marbles did not exhibit obvious shrinkage after the complete evaporation of both oil and water phases. Three reasons were proposed to explain the successful fabrication of Pickering emulsion marbles: (1) The increment of stabilizer particles made the emulsion much more stable, which inhibited the aggregation of water droplets during the vaporization of CH_2_Cl_2_; (2) The increment of stabilizer decreased the size of emulsion droplets, slowing down the evaporation of CH_2_Cl_2_; (3) The dissolved PLLA would separate out and sequentially adhere to the surface of emulsion droplets, impeding the accumulation of a disperse phase. It must be pointed out that excessive PLLA in CH_2_Cl_2_ solution (P_0.04-0.12-2_) or exorbitant internal phase volume fraction (P_0.04-0.1-3_) would make the formed Pickering emulsion too viscous to be injected drop by drop from the needle (Figure S2A), which is adverse for the fabrication of spherical emulsion marbles.

Pure CH_2_Cl_2_ liquid marbles containing PLLA were also prepared, for comparison (Figure 2A,A_1_). It was clear that the CH_2_Cl_2_ liquid marble underwent severe shrinkage after drying while Pickering emulsion marbles kept their initial shape (Figure 2B,B_1_). This phenomenon demonstrated that the Pickering emulsion marbles successfully inhibited the shrinkage of PLLA film during the solution vaporization. In addition, the size of Pickering emulsion marbles was much larger than that of CH_2_Cl_2_ liquid marbles, which was attributed to the increasing viscosity of emulsion compared with CH_2_Cl_2_ solution. It should be noted that the Pickering emulsion droplet was rolled in a petri dish containing H_30_ silica particles until its surface was completely covered with powder (Figure S2B). The average amount of silica particles in one capsule was estimated to be 0.08 mg.

**Figure 3:**
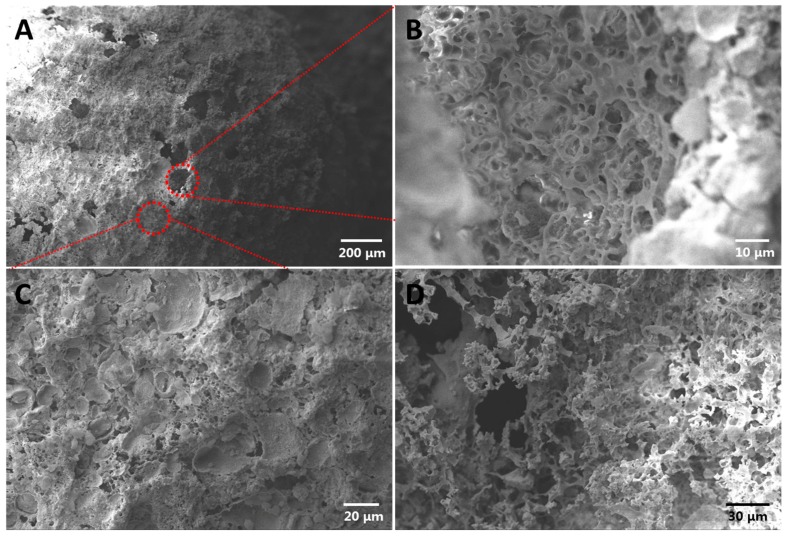
SEM images of prepared cellular capsule derived from Pickering emulsion-based marbles (P_0.02-0.1-2_): (**A**) Overall morphology of prepared cellular capsules; (**B**) Internal cellular structure of the capsule from the perspective of large hole distributing on the surface; (**C**) Surface morphology of the capsule; (**D**) Inner structure of the capsule after incision.

The technique of SEM was employed for interrogating the microstructure and morphology of the prepared cellular capsules deriving from Pickering emulsion marbles. Figure 3A shows the overall morphology of capsules. Holes with a diameter of approximately 80–100 μm were distributed on the capsule surface. Figure S5 helps to explain this phenomenon: During the drying process, the CH_2_Cl_2_ liquid layer between Pickering emulsion droplets (especially for large droplets near the marble surface) and marble surface was not thick enough, and the separated PLLA film after the complete vaporization of CH_2_Cl_2_ was too thin to cover the whole surface of the droplets, resulting in the generation of holes on the surface of the capsule. In addition, considering that the evaporation rate of CH_2_Cl_2_ was much faster than that of water, the water droplets still existed after the complete vaporization of CH_2_Cl_2_, and the surface tension from those droplets facilitated the formation of holes. Moreover, the diameter of the hole was about 50 μm, and this value was identical to the size of some Pickering emulsion droplets in Figure 1B, which supported the proposed explanation. We found a cellular structure inside of the capsule (Figure 3B), which was identical to our expectation. To further confirm the internal structure, we cut the capsule into slices and characterized them with SEM (Figure 3D). It was obvious that the inner morphology of the capsule was a cellular structure, which was consistent with the conclusion coming from Figure 3B. Figure 3C is the local enlarged image of the capsule surface. It was clear that the capsule surface was a porous morphology at high magnification, though they were relatively flat at low magnification. This phenomenon could also be attributed to Pickering emulsion templates.

### 3.3. In Vitro Drug Release

**Figure 4:**
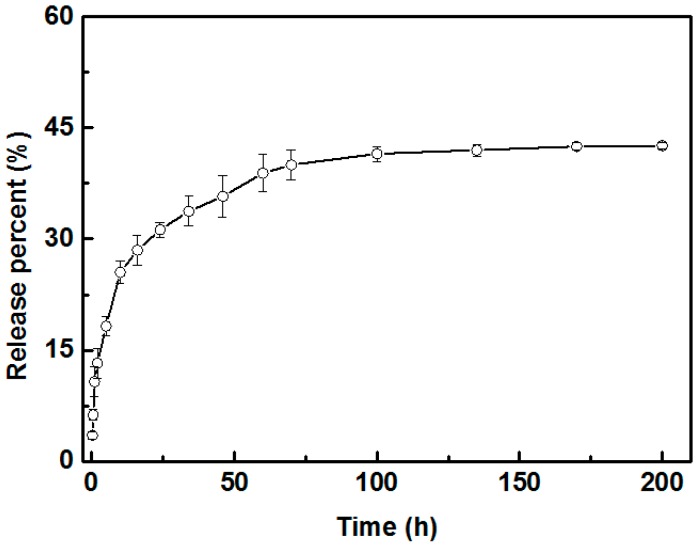
In vitro release curve of cellular capsule deriving Pickering emulsion marbles.

Many substances, such as hydrogels [23] and particles [24], have been employed as vehicles for the encapsulation and sustained release of the drug in biological territory. The porous PLLA scaffold [25], which has proved to be a preferred carrier for sustained drug release inspired us to attempt a similar application of the prepared cellular capsules. The enrofloxacin, a common bactericide for animals, was exploited for exploration of the sustained drug release of prepared cellular capsules. Figure 4 shows the in vitro release curve of enrofloxacin encapsulated in capsule scaffold. It can be seen that the enrofloxacin was released relatively quickly during the initial period of 50 h, and the release percentage was up to 36.0%. After this period, the release rate of enrofloxacin from capsules became slow until the release percent reached a stable value (about 42%). After 100 h, the release curve did not show any more apparent fluctuation, suggesting the drug release became steady with a final value of 42%. In conjunction with the biocompatibility of PLLA and H_30_ silica particles, the prepared cellular capsules could be a promising candidate for the application of sustained drug release.

## 4. Conclusions

In this report, we prepared Pickering emulsion-based marbles and fabricated the cellular capsules from the prepared emulsion marbles via facile vaporization of both oil and water phases in ambient atmosphere. The solution of PLLA in CH_2_Cl_2_ was utilized as the oil phase of Pickering emulsion, and H_30_ silica particles served as both stabilizer for Pickering emulsion and enwrapping powder for emulsion marbles. Influences on the formation of emulsion marbles were explored in detail and it was concluded that favorable stability and appropriate viscosity of the emulsion would facilitate the fabrication of emulsion marbles. The morphology of the obtained cellular capsules was characterized by SEM and the result was identical to our expectation. In addition, the test of in vitro drug release using enrofloxacin as a model showed that the fabricated capsules impart favorable property of sustained drug release, which make it a potential candidate in the application of biomedical territory combining the excellent biocompatibility and biodegradability of PLLA.

## Figures and Tables

**Table 1:** Recipes of the different Pickering emulsion.

Serial	H_30_ (w/v %) ^a^	PLLA (w/v %) ^a^	CH_2_Cl_2_ (mL)	Water (mL)
P_0-0.1_	0	5	2	0
P_0.01-0.1-2_	0.5	5	2	2
P_0.02-0.1-2_	1.0	5	2	2
P_0.04-0.1-2_	2.0	5	2	2
P_0.04-0.12-2_	2.0	6	2	2
P_0.04-0.1-3_	2.0	5	2	3

**^a^** With respect to oil phase.

## Supplementary Materials

The supplementary materials are available online at www.mdpi.com/1996-1944/9/7/572/s1.
