# Supplementary material for: Pickering Emulsion-Based Marbles for Cellular Capsules
Source: Materials (Basel). 2016 Jul 14;9(7):572. doi: 10.3390/ma9070572 (PMC5456839; doi:10.3390/ma9070572)
Supplement: Supplementary file 1 [file materials-09-00572-s001.pdf]

Supplementary Materials: Pickering Emulsion-Based Marbles for Cellular Capsules

Guangzhao Zhang and Chaoyang Wang

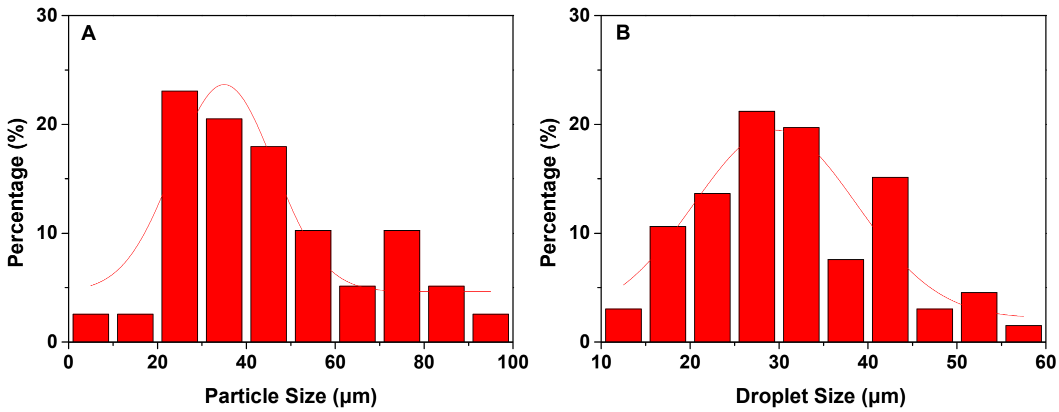

Figure S1. Pickering emulsion droplets size distribution with different stabilizer content: (**A**) 1 w/v % of H_{30} silica particles, sample $P_{0.02\text{-}0.1\text{-}2}$; (**B**) 2 w/v % of H_{30} silica particles, sample $P_{0.04\text{-}0.1\text{-}2}$.

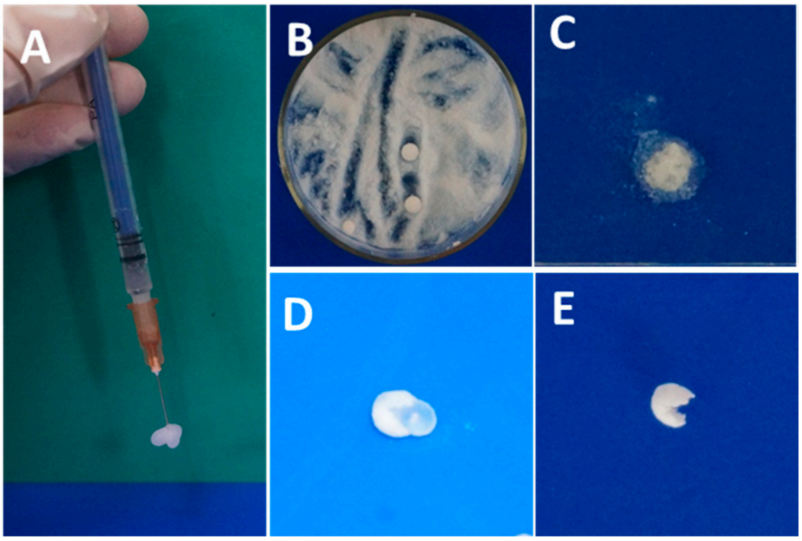

Figure S2. (**A**) the Pickering emulsion was too viscose to be injected dropwise from needle when the concentration of PLLA or stabilizer particles was too high; (**B**) The preparation of Pickering emulsion marbles via rolling with silica particles; (**C**) the Pickering emulsion marbles break after transferring to a glass base when the concentration of stabilizer particles was set to 0.5 w/v % (sample $P_{0.01\text{-}0.1\text{-}2}$); (**D**) water phase was extruded out from emulsion marbles when the concentration of stabilizer particles was 1 w/v % (sample $P_{0.02\text{-}0.1\text{-}2}$); (**E**) the formative capsule after dry from marbles in Picture (**D**).

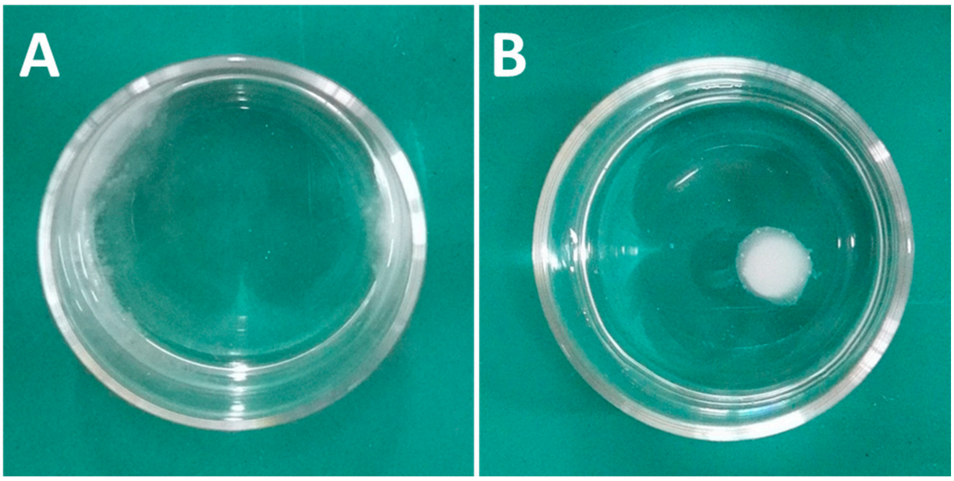

Figure S3. Type determination of water in oil Pickering emulsion: (**A**) in CH_2Cl_2 solution; (**B**) in ultrapure water.

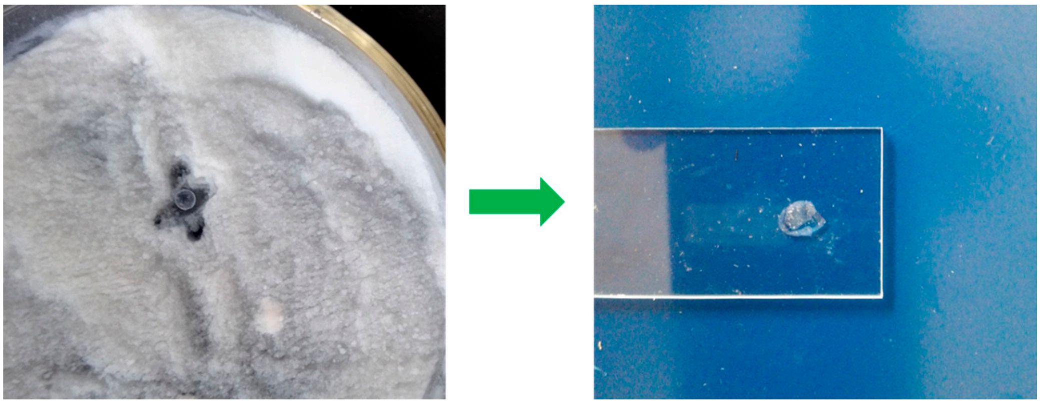

Figure S4. The attempt of utilizing pure water to prepare liquid marble with H_{30} particles.

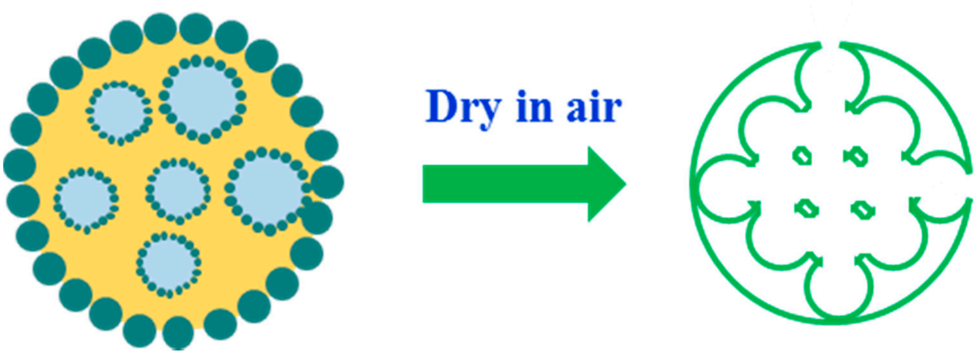

Figure S5. The formation mechanism of pores on the surface of capsule.
